# Self-assembly and wetting properties of gold nanorod–CTAB molecules on HOPG

**DOI:** 10.3762/bjnano.10.69

**Published:** 2019-03-13

**Authors:** Imtiaz Ahmad, Floor Derkink, Tim Boulogne, Pantelis Bampoulis, Harold J W Zandvliet, Hidayat Ullah Khan, Rahim Jan, E Stefan Kooij

**Affiliations:** 1 Physics of Interfaces and Nanomaterials, MESA+ Institute for Nanotechnology, University of Twente, P. O. Box 217, 7500AE Enschede, The Netherlands; 2 Department of Physics, University of Peshawar, Khyber Pakhtunkhwa 25120, Pakistan; 3 School of Chemical and Materials Engineering, National University of Sciences and Technology, Islamabad 44000, Pakistan

**Keywords:** CTAB, gold nanorods, micelles, self-assembly, wettability

## Abstract

The formation of self-assembled superstructures of cetyltrimethylammonium bromide (CTAB) after drying on a nonwetting highly ordered pyrolytic graphite (HOPG) surface have been investigated using scanning electron microscopy (SEM) and atomic force microscopy (AFM). Although SEM did not reveal coverage of CTAB layers, AFM showed not only CTAB assembly, but also the dynamics of the process on the surface. The self-assembled layers of CTAB molecules on the HOPG terraces prior to nanorod deposition were shown to change the wettability of the surface, and as a result, gold nanorod deposition takes place on nonwetting HOPG terraces.

## Introduction

In the last few decades there have been many studies that focused on hard colloidal particles and micelles, covering a length scale ranging from nanometers to micrometers. The focus of nanoscience and nanotechnology is increasingly shifting from synthesis to assembly into larger superstructures. Although these materials show significant optical and electronic properties [1–3], the structural correlation between the deposited nanomaterial and where they are deposited on the surface is also of fundamental importance [4–7]. Additionally, owing to its simplicity, versatility and low cost, the process of self-assembly at the liquid–solid interface has proved to be an attractive self-assembly route [4,8–9]. The self-assembled structures can play an important role in magnetic [10–13], electronic [14–16], photovoltaic [17–19], biomedical [20–22], sensing [23–25], catalytic [26–29], photonic [30–34], plasmonic [35–39], and surface-enhanced Raman scattering (SERS) [40–42] applications.

In relation to our system consisting of cetyltrimethylammonium bromide (CTAB)-coated gold nanoparticles, it has also been observed that CTAB surfactant molecules can self-assemble on a highly ordered pyrolytic graphite (HOPG) surface in the form of hemi-cylindrical micelles [43–45]. The surface of HOPG is hydrophobic [46–47], while the CTAB molecules have a hydrophilic end group and a hydrophobic tail [48]. Therefore, to shield their hydrophobic tail from the water phase, these molecules form spherical and cylindrical micelles in aqueous suspension [49–50]. Xu et al. were the first to observe the adsorption of CTAB molecules on HOPG in head-to-head arranged layers [50]. It has recently been reported that CTAB-stabilized gold nanorods align with the step edges whereas side-by-side close packed arrays oriented in all possible directions were observed at the terraces on HOPG [51]. In their work, however, the notable role of CTAB on a HOPG surface is not highlighted.

It has been reported [43,45] that on nonpolar surfaces such as HOPG, CTAB molecules assemble in the form of hemi-cylindrical micelles. Manne et al. [52] proposed a model for the deposition of CTAB molecules on HOPG at various concentrations. At low concentration (≈10% of the critical micelle concentration), the molecules adsorb with their alkane chains extended along the substrate plane. Such a configuration of CTAB molecules oriented parallel to the HOPG surface has been observed experimentally [53], where van der Waals interactions are dominant between molecules and the substrate.

The main focus of the present work is to investigate CTAB superstructures on HOPG substrates using atomic force microscopy (AFM). The assembly of CTAB molecules was investigated at various positions on the substrate. Also, the role of CTAB molecules that changes the wettability of the HOPG terraces is discussed in relation to the previous work [51]. The application of such studies could be relevant especially in those areas where conversion of a nonwetting surface (or some particular regions of a surface) to a wetting ones is desired. Also, the presence of such CTAB layers (which cannot be observed using scanning electron microscopy (SEM)) between the substrate and assembled nanorods can disturb the desired properties between the associated gold nanorods and the HOPG surface. Furthermore, the study and understanding of various assembled morphologies of surfactant molecules is relevant in many other areas [13–14,32].

## Results and Discussion

**Figure 1:**
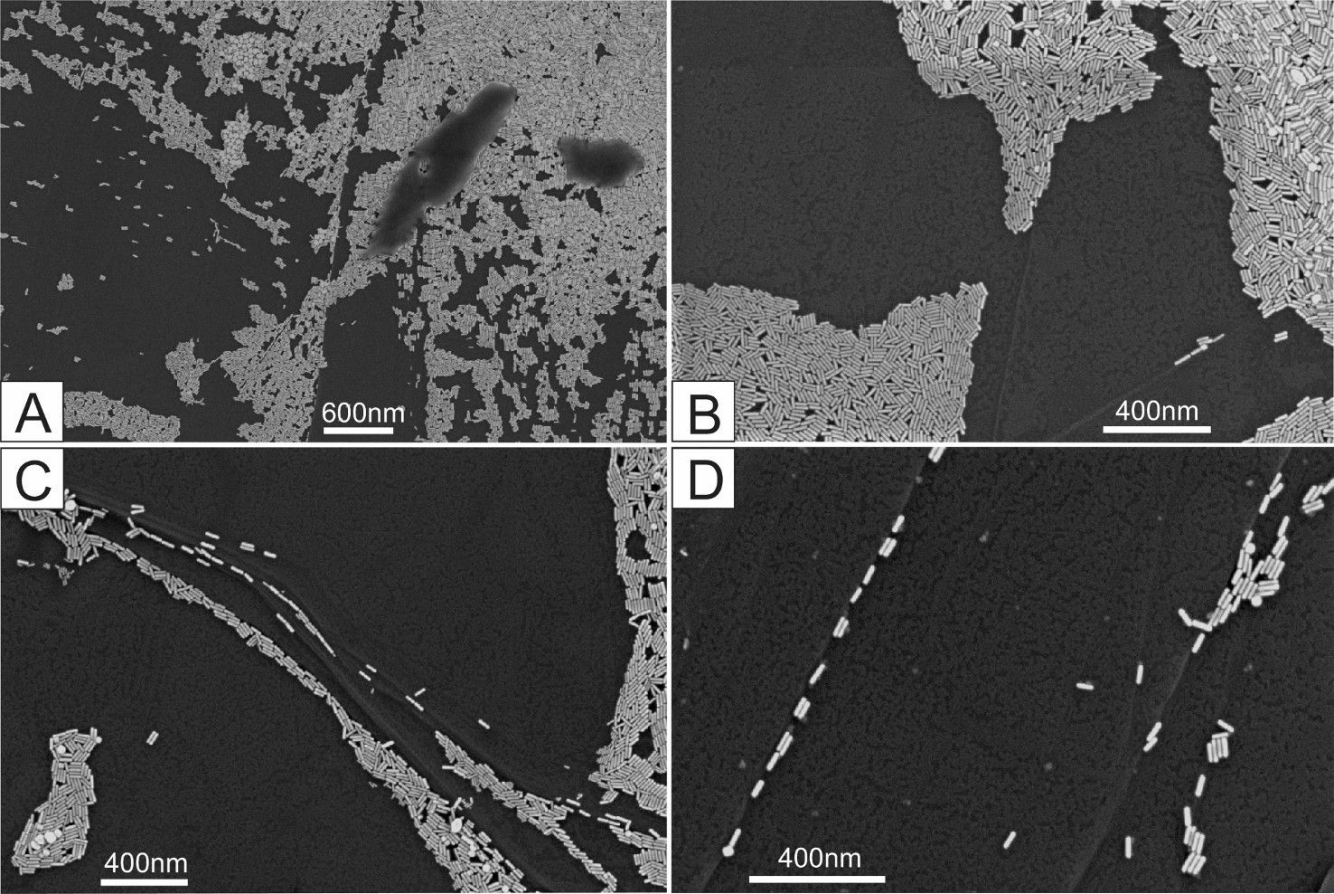
SEM images of evaporation-induced deposits at various locations on the HOPG surface: (A) dense deposits of gold nanorods after the first pinning of the contact line; (B) monolayer close-packed arrays on terraces; and (C, D) nanorods deposits at the step edges.

In drying experiments on nonwetting substrates, suspension droplets typically leave a deposit of particles after evaporation of the solvent in the form of a cluster [54–56]. For instance, to maintain its contact angle on nonwetting surface like HOPG, the inward motion of the shrinking droplet will sweep away all particles from the HOPG terraces except those that settle on the wall of the step edges. Consequently, a cluster of deposited particles will be observed in the end whereas other areas of the substrate will be free from such particles. Thus, in principle, no particles should be found on the nonwetting HOPG terraces. However, Figure 1A–C displays a montage of SEM images that shows monolayer nanorod deposits on the HOPG terraces, analogous to the results reported previously [51]. Since this work was more focused on the deposition and alignment of the nanorods near the step edges (comparable to the observation illustrated in Figure 1C,D), deposition on the nonwetting HOPG terraces could not be explained. In the sections that follow, our focus in this work will be on the HOPG terraces. Here we will highlight the nanorod deposition on the HOPG terraces that is assisted by hidden features (in SEM images) of the self-assembled CTAB molecules.

As a cationic surfactant, CTAB molecules consist of a hydrophilic head and a hydrophobic tail. In an attempt to shield the hydrophobic tail from the aqueous phase, CTAB molecules assemble on HOPG in the form of hemi-cylindrical micelles [57] and cover the entire region occupied by the droplet. As such the wettability of the HOPG surface was transformed by establishing a hydrophilic layer underneath the nanorods.

**Figure 2:**
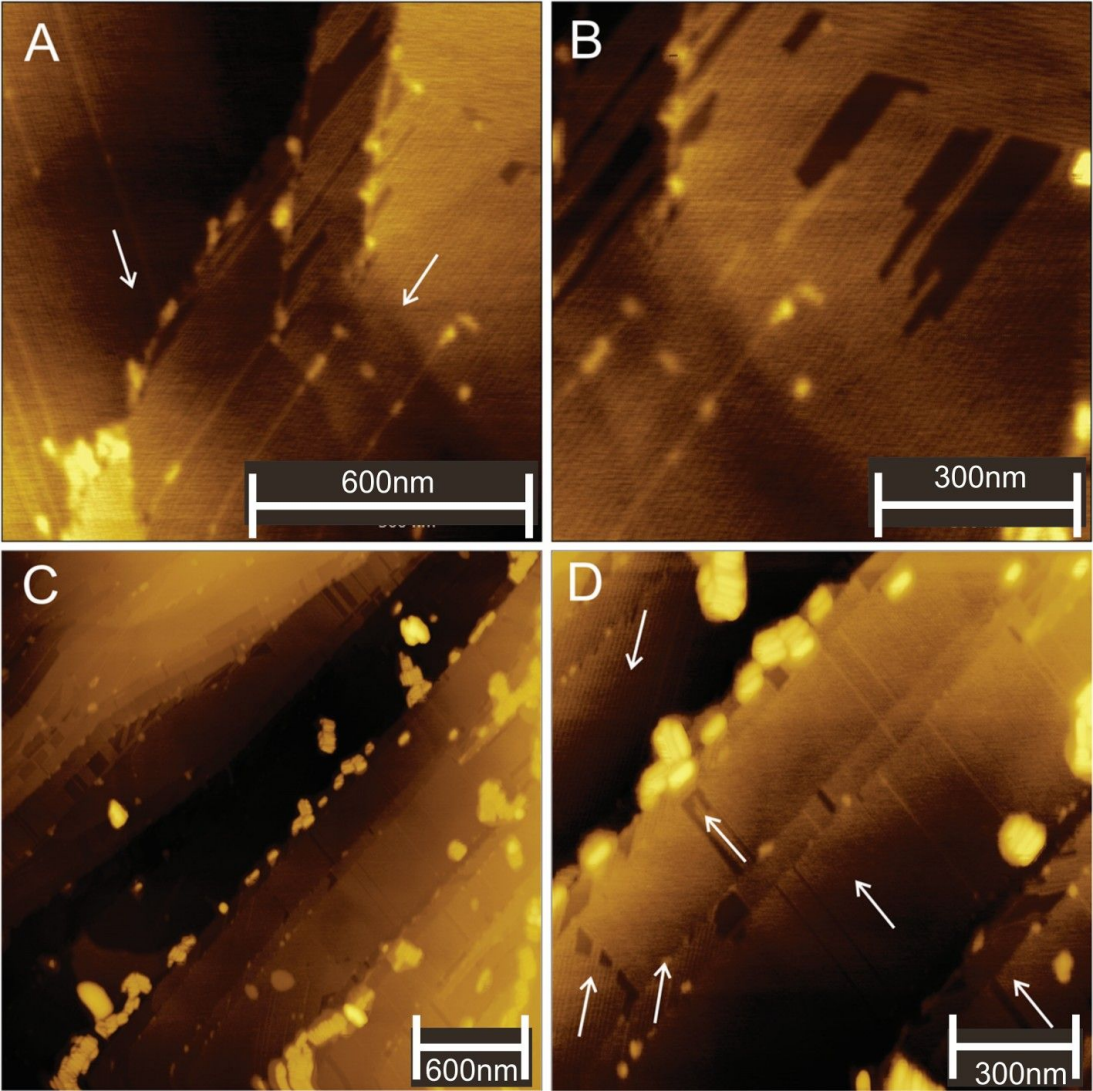
(A, C) Overview and (B, D) detailed AFM images of gold nanorods deposited in the CTAB surrounding at the step edges and terraces of the HOPG surface. White arrows indicate the direction of the stripes.

In Figure 2 AFM images reveal CTAB stripes oriented in two specific directions, as indicated by the arrows. The angle between these directions is 60° or 120°, as shown in Figure 2A,D. The brighter regions are gold nanorods found preferentially at the step edges. The relatively dark areas in Figure 2B indicate regions devoid of stripes. This effect is highlighted in the following paragraphs.

As previously mentioned, SEM (Figure 1) and AFM (Figure 2) images revealed complimentary results: where SEM cannot be used to ascertain some important self-assembled CTAB topographies, AFM reveals the hidden traces of CTAB molecules all over the HOPG surface. For the first time, to the best of our knowledge, new features of CTAB molecular arrays are also observed on the HOPG terraces, as described in the following.

### Islands

**Figure 3:**
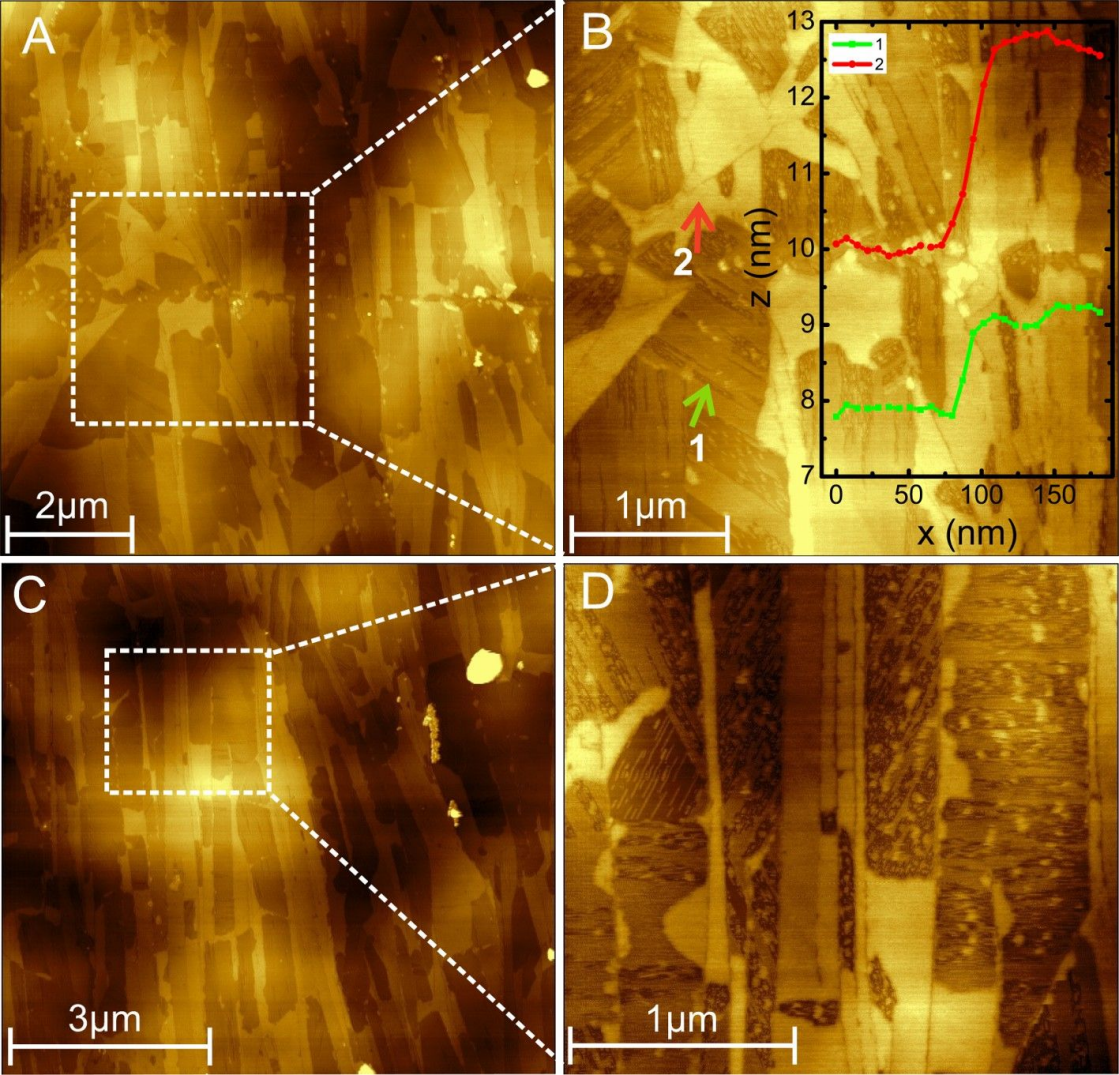
AFM images of CTAB islands formed on various terraces of HOPG. (A) Overview of the islands and (B) enlarged image of the white square region in (A); the inset shows the line scans indicated by 1 (green) and 2 (red). (C) Another overview of the CTAB islands, and (D) enlarged view of the region enclosed by the white square in C.

The brighter areas in the AFM images (Figure 3A) correspond to the various HOPG terraces decorated with CTAB deposits; nevertheless, they do not all demonstrate the characteristic 60° or 120° angles typically observed for HOPG. Instead they indicate islands with round peripheries, as shown in Figure 3B–D. This could be due to the fact that the deposited layers of CTAB (on top of each other) do not have an effect similar to the bare HOPG. The height profiles in the inset of Figure 3 indicate that the islands exhibit different heights; the smallest step size is ≈1 nm, which could originate from a single layer of CTAB, while the larger step (red curve) is ≈3nm, indicating multiple CTAB layers on top of each other.

### Stripes

**Figure 4:**
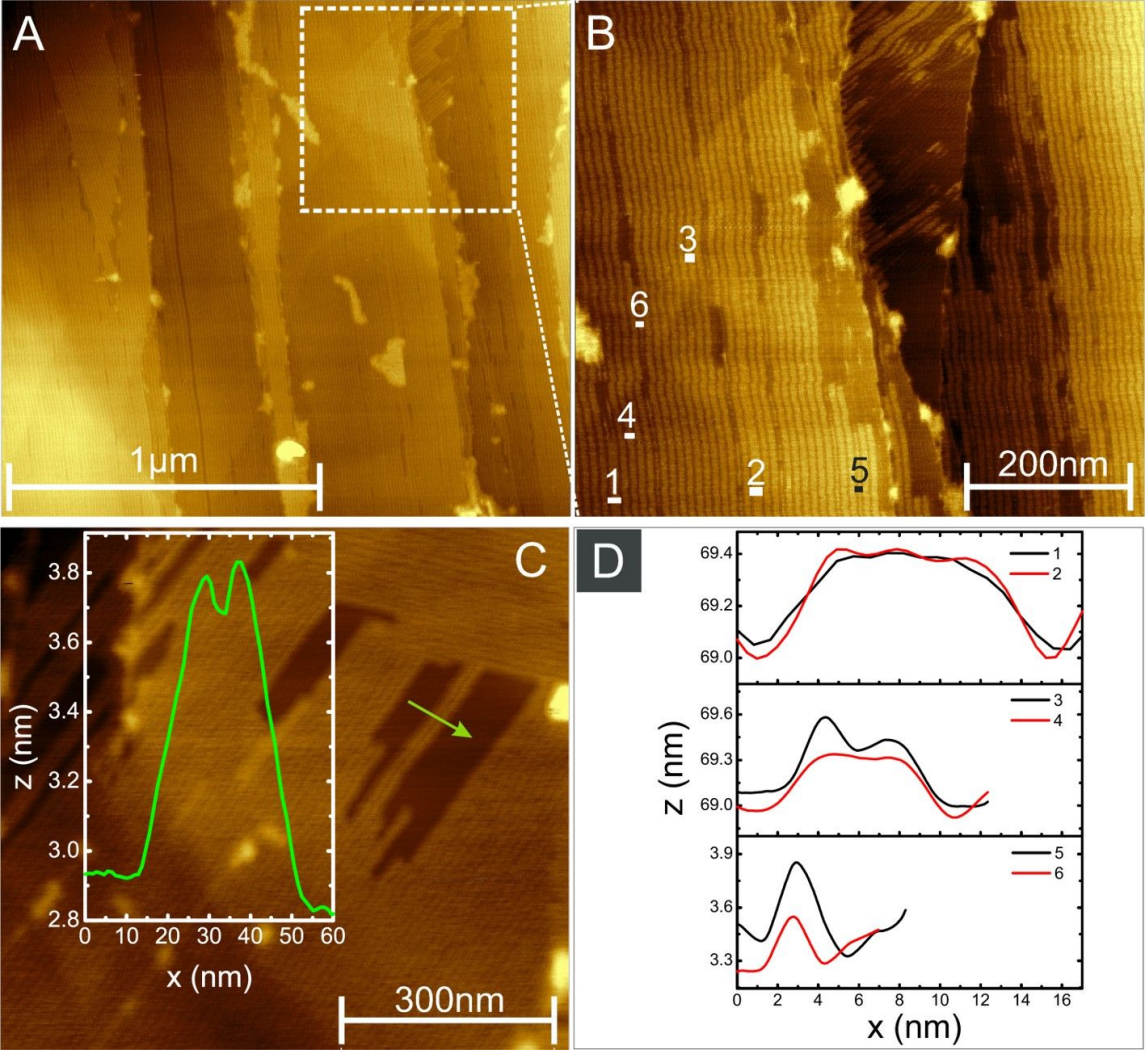
Self-assembled CTAB molecules on the HOPG surface imaged using AFM. (A) Overview of CTAB self-assembled stripes. (B) Enlarged view of the white square region in A, revealing the characteristic stripes. (C) Stripes of CTAB with a height profile along the green line. (D) Stripe widths at various locations in B indicated by the number 1–6.

Within the CTAB islands, stripe-like arrangements of CTAB molecules can be clearly seen on the terraces of the HOPG, Figure 4A–C. Figure 4A shows an overview of linearly arranged CTAB stripes on various HOPG terraces. The height profiles, Figure 4D, suggest that the stripes have three different widths with values of 12 nm, 8 nm, and 2 nm. Most of the stripes were of ≈8 nm width. The measured height of the stripes was ≈0.4 nm, Figure 4D. This value, however, does not reflect the true height of CTAB. This is due to convolution effects related to the inter-stripe gap (gap width ≈2.5 nm), which is much smaller than the radius of curvature of the AFM tip (≈8 nm).

A more accurate estimation of the height of the CTAB stripes can be obtained from height profiles of isolated stripes next to the regions where the bare substrate is exposed (Figure 4C). From the corresponding profile, the actual height was determined to be ≈0.8 nm. These calculated values are smaller compared to the CTAB molecules (length ≈1.5–2 nm) reported previously [57–58]. This discrepancy suggests that the molecules do not adsorb in a conformation perpendicular to the substrate. Instead, they may be compressed or, more likely, tilted with respect to the surface normal.

### Stripe dynamics

**Figure 5:**
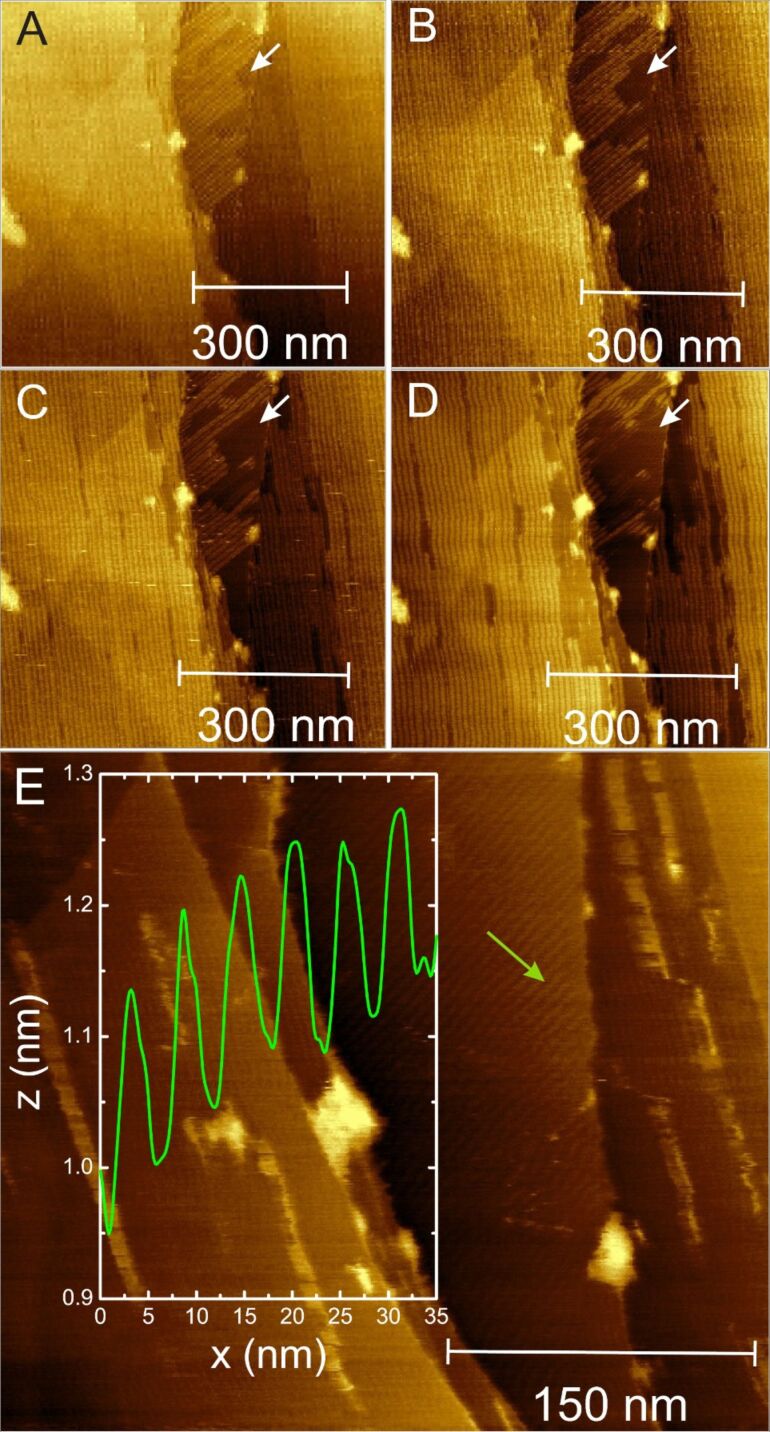
(A–D) Sequential AFM images depicting the dynamics of self-assembled CTAB stripes on terraces; for reference, a defect which acts as a vacancy nucleation site is indicated by the white arrows. (E) After the large stripes have completely disappeared, a corrugation of the surface is still observed; the inset depicts the gap width (≈2.5 nm), height (≈0.15 nm), and width (≈3 nm) of the residual striped features.

Sequentially acquired AFM images, Figure 5, display interesting dynamics of the self-assembled stripes. The time delay for the first four images, Figure 5A–D, was ≈4 min between any two consecutive images, and ≈10 min for Figure 5D to Figure 5E. Close examination of the left portion of the images, Figure 5A–D, revealed that the stripes on the terrace appear stable for a long time, whereas the ones on the right portion begin to disappear. We ascribe this disappearance of the stripes to originate from the dislocation of CTAB molecules, creating point defects. Such defects will make the molecules less tightly bound at the defect site, and hence, support the growth of defects.

As indicated, the CTAB stripes seem to disappear with time in the regions where the surface morphology is probed using AFM. From all these images, however, we cannot conclusively determine the origin of the removal rate of the stripes. Zooming out does not reveal molecules piling up at the edges of the scan region. In addition, protruding surface features represented by the brighter spots in the AFM images, Figure 5, do not seem to grow significantly in size. Owing to the limited scan range, we also cannot explicitly claim that the molecules desorb from the surface.

**Figure 6:**
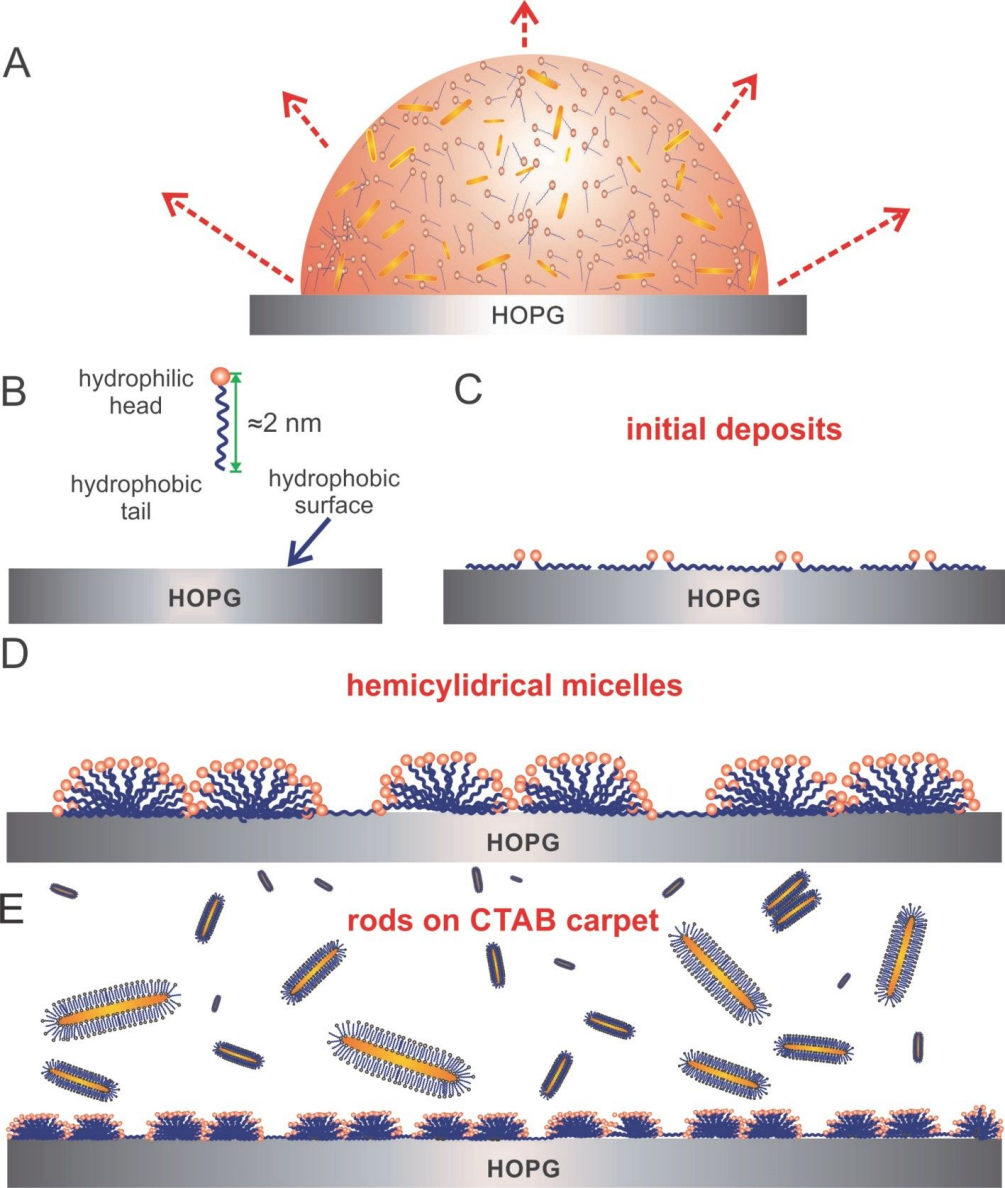
(A) Schematic representation of a droplet containing CTAB-coated nanorods and a surplus of CTAB on the HOPG surface; the arrows indicate the relative evaporation rate of the solvent. (B) Hydrophobic HOPG surface and amphiphilic CTAB molecule. (C) Initially adsorbed molecules will deposit flat on the surface to maximize the interaction between the hydrophobic tail and the nonpolar substrate. (D) Subsequent adsorption of molecules will lead to the formation of hemi-cylindrical micelles. (E) Nanorods assemble on the exposed hydrophilic CTAB layer.

Further analysis of the AFM images following a prolonged scanning of the surface leads to the intriguing observation that after complete removal of the aforementioned stripes, the exposed surface still exhibited a periodic structure. A typical example is shown in Figure 5E; the inset represents a line scan (green arrow) across the lower stripes. Surprisingly, this striped phase has an even more regular structure, with stripes of ≈0.15 nm high and separation width ≈2.5 nm. As such, the width of each stripe is ≈3 nm and composed of a single molecular array adsorbed parallel to the surface (Figure 6C). Such an arrangement differs from that described in Figure 4D where these molecules adsorb in the form of hemicylindrical micelles, also modeled in Figure 6D. Nevertheless, the orientation of upper and lower stripes is similar.

The CTAB molecule has a length of 1.5–2 nm [53,57–58]. This agrees well with the diameter of spherical CTAB micelles (4.4 nm), being approximately twice the CTAB length. In principle, a molecule oriented perpendicular to the surface (there with a height of the hemi-cylindrical micelles, Figure 6) should be ≈2 nm (height of fully stretched CTAB molecule). The height of the self-assembled CTAB stripes, however, are markedly lower, typically ≈0.8 nm. This lower height value can also be attributed to the molecules being compressed or twisted and skewed upon evaporation of the solvent, thereby giving rise to shorter features, as indicated by the AFM data.

### Nanorod deposits over CTAB layers

**Figure 7:**
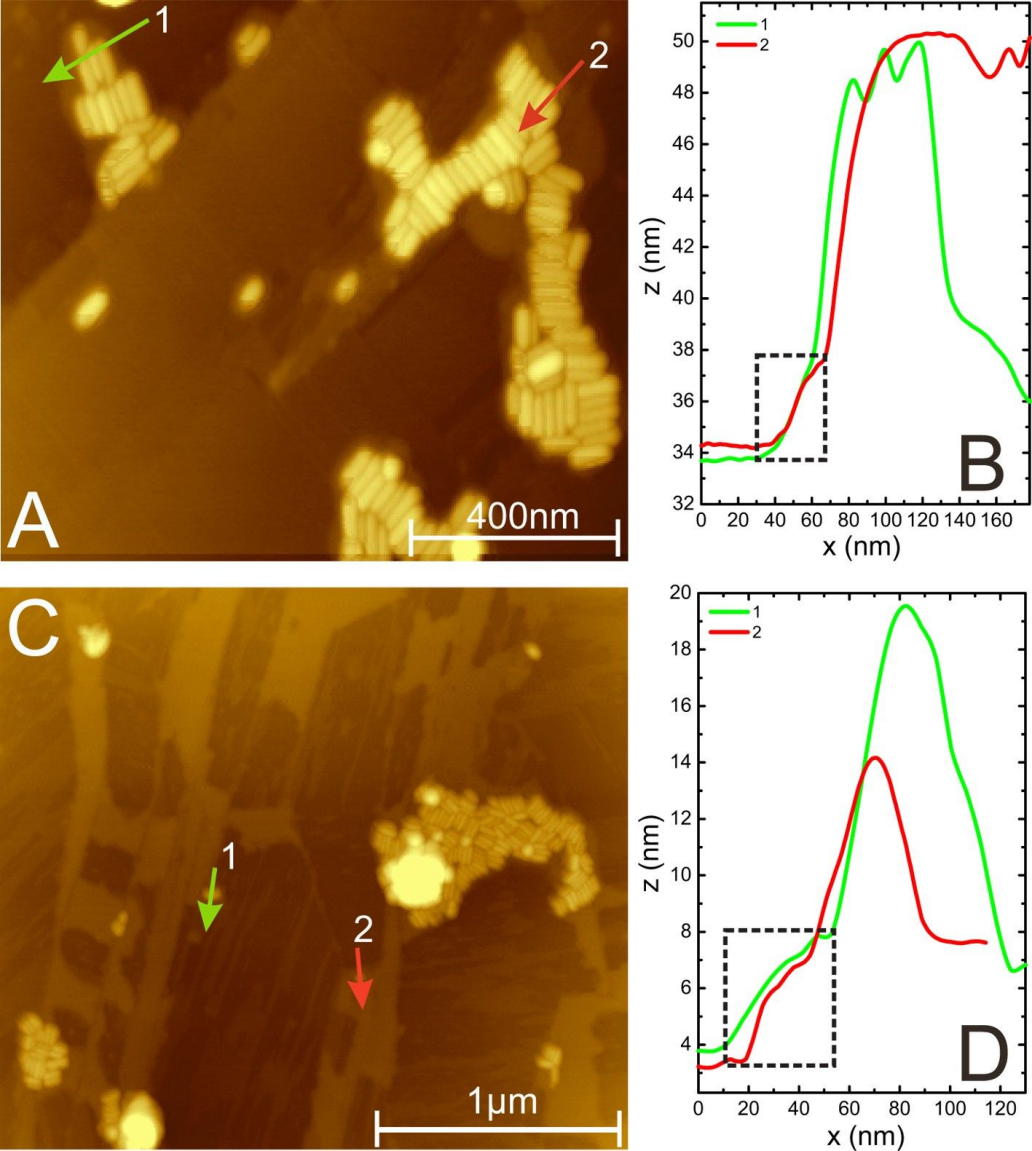
(A,C) Gold nanorod deposits on CTAB-coated HOPG. (B,D) Height profiles of green and red arrows in (A) and (C) are plotted on the right side of the AFM images. The dashed rectangles indicate the CTAB layer height underneath the nanorod deposits.

In the above sections, it has been discussed in detail that the presence of CTAB molecules in a gold nanorod suspension will self-assemble all over the HOPG surface as shown in Figure 3, Figure 4, and Figure 5. Such deposition of CTAB layers on top of HOPG terraces will permit nanorods close to the three-phase contact line to deposit on the HOPG terraces. For instance, Figure 7 shows AFM images of deposited layers of CTAB molecules underneath the gold nanorod deposits on the HOPG terraces. This shows that surfactant-coated nanorods, with their hydrophilic end groups exposed, do not prefer to deposit on bare hydrophobic terraces of the HOPG. This is consistent with our initial premise that during predeposition, CTAB molecules first self-assemble on the surface due to their abundance in the solvent. On the bare HOPG surface, one can expect that assembled arrays of gold nanorods in suspension within the droplet will sweep the nanorods inward during evaporation via slipping of the three-phase contact line during inward motion. However, CTAB assembled morphologies on the surface prevent this from happening as the surface become wet (hydrophilic) with the adsorption of CTAB on the HOPG surface. Consequently, the wet (CTAB stabilized) surface of the gold nanorods will deposit over those regions on the HOPG substrate, which are more hydrophilic than the bare HOPG. As a result, nanorod deposits are only found in those regions of the substrate where CTAB was already deposited. The line scan 1 (green) and 2 (red) of Figure 7A and 7C are shown in Figure 7B and 7D, respectively. Noticeably, Figure 7A and 7C show the region of assembled gold nanorods on top of the CTAB deposits (height ≈2.5 nm) as depicted by the dashed rectangular regions in Figure 7B and 7D, respectively. Furthermore, the height of CTAB in Figure 7B and 7D is ≈2.5 nm, whilst Figure 4C reveals a much smaller height of ≈0.8 nm. The possible reason for such a discrepancy in the CTAB height can be attributed to the multiple layers of CTAB on the surface, as evident in the inset of Figure 3.

## Conclusion

In this manuscript we present results on the assembly of CTAB molecules and deposits of gold nanorods on HOPG surfaces. An aqueous droplet containing gold nanorods stabilized with CTAB surfactant was allowed to evaporate. After complete evaporation of the solvent, the deposits were analyzed using SEM and AFM. The assembly of gold nanorods on the HOPG surface was similar to what was reported in previous work. Notably, in contrast to AFM, SEM was unable to detect CTAB features on the surface. The AFM images reveal a striped phase on the HOPG surface, which we ascribe to self-assembled CTAB layers. The observed stripes are oriented along the typical substrate features on a micrometer length scale. Careful analysis of the stripe dimensions, i.e., width, height and spacing, suggest that the stripes consist of multiple hemi-cylinders separated by a distance corresponding to the length of a single CTAB molecule. With increasing scan time, defect sites are observed, which develop following the vacancy regions, and grow as a function of time on various HOPG terraces. Self-assembled layers of CTAB changed the wettability of the surface and facilitated the deposition of gold nanorods on HOPG.

## Experimental

Some of the experimental protocols and conceptual framework used in this work are similar to our work presented elsewhere [9,51].

### Materials

Hydrogen tetrachloroaurate (HAuCl_4_·3H_2_O, 99.999%, Aldrich), silver nitrate (AgNO_3_, 99%, Acros), ascorbic acid (AA, 99%, Merck), cetyltrimethylammonium bromide (CTAB, Aldrich, 98%), sodium borohydrate (NaBH_4_, 99%, Aldrich), hydrochloric acid (HCl, 37%, Merck), (3-aminopropyl)triethoxysilane (APTES, 99%, Acros), and sodium citrate (99%, Aldrich) were all used as received without further purification. The water used in the synthesis was of Milli-Q quality (18.2 MΩ cm), produced in a Simplicity 185 system (Millipore).

### Synthesis

A seed-mediated procedure was employed, as described by Nikoobakht and El-Sayed [59], to synthesize the gold nanorods. CTAB-coated seed particles were prepared by mixing 25 µL of HAuCl_4_ (0.1 M) with 10 mL of CTAB (0.1 M). 60µL of ice cold NaBH_4_ (0.1 M) was added and the mixture was continuously stirred for 3 min. The mixture quickly turned light brown, indicating the formation of gold seeds. The solution was kept unperturbed at room temperature (25 °C) for two hours.

To synthesize gold nanorods with an aspect ratio (AR) of ≈3, the growth solution was prepared by introducing 50 µL of HAuCl_4_ (0.1 M) in 10 mL of CTAB (0.1 M). The solution was kept at 30 °C for 20 min at slow stirring to dissolve CTAB and then it was cooled down to 25 °C. At this temperature (25 °C), 25 µL of AgNO_3_ (0.1 M) was introduced, followed by 70 µL of AA (0.1 M); the subsequent solution turned colorless. This was followed by addition of 100 µL of HCl (1 M) to maintain the pH of solution at 3. Finally 24 µL of the seed solution was added and the resulting solution was left undisturbed overnight at room temperature.

### Separation of byproduct

The as-prepared growth solution was centrifuged twice at 15000 rpm for 10 min to reduce (1.0 mM to ≈0.4 mM) the excess CTAB concentration. Similarly, the same solution was centrifuged again at 5600 rpm for 5 min to separate nanospheres from nanorods. The supernatant, consisting mostly of nanorods, was carefully separated from nanospheres stuck at the bottom of the centrifuge tube.

### Characterization techniques

Scanning electron microscopy (SEM) imaging of the samples was undertaken with a Zeiss 1550 system (optimum resolution ≈1 nm at 2 kV accelerating voltage). Tapping-mode AFM imaging was carried out with an Agilent 5100 atomic force microscope using HQ:NSC35/Al probes (Mikromasch) with a nominal spring constant of 5–16 N/m and a resonance frequency 150–300 kHz.

### Drop casting

A 5 μL droplet of gold nanorod suspension was deposited on a freshly cleaved HOPG surface and the solvent was left to evaporate at room temperature. After 2 h, the solvent was completely evaporated leaving behind bright regions of deposits on the surface.
